# Metabolic Disturbances in Rat Sublines with Constitutionally Altered Serotonin Homeostasis

**DOI:** 10.3390/ijms22105400

**Published:** 2021-05-20

**Authors:** Maja Kesić, Petra Baković, Ranko Stojković, Jasminka Štefulj, Lipa Čičin-Šain

**Affiliations:** 1 Department of Molecular Biology, Ruđer Bošković Institute, HR-10000 Zagreb, Croatia; Maja.Kesic@irb.hr (M.K.); Petra.Bakovic@irb.hr (P.B.); 2 Division of Molecular Medicine, Ruđer Bošković Institute, HR-10000 Zagreb, Croatia; Ranko.Stojkovic@irb.hr

**Keywords:** serotonin, obesity, metabolism, genetic rat model, adipose tissue

## Abstract

Central and peripheral serotonin (5HT) have opposing functions in the regulation of energy homeostasis. Both increasing 5HT signaling in the brain and decreasing 5HT signaling in the periphery have been proposed as potential treatments for obesity. This study investigates the relationship between constitutionally high or low 5HT activity and systemic net energy balance. Two sublines of rats with high and low whole-body 5HT tone, obtained by selective breeding for platelet 5HT parameters, were examined for fat accumulation in different white adipose tissue (WAT) depots, glucose/insulin tolerance, blood metabolic parameters, and expression of various metabolic genes. High-5HT animals, unlike their low-5HT counterparts, developed widespread intra-abdominal obesity associated with glucose and insulin intolerance, which worsened with age. They also had elevated blood glucose and lipid parameters but showed no significant changes in circulating leptin, resistin, and adipsin levels. Surprisingly, adiponectin levels were increased in plasma but reduced in the WAT of high-5HT rats. A limited number of metabolic genes belonging to different functional classes showed differential expression in WAT of high-5HT compared to low-5HT rats. Overall, a constitutive increase in 5HT tone is associated with a positive energy balance acting through subtle dysregulation of a broad spectrum of metabolic pathways.

## 1. Introduction

The maintenance of energy homeostasis is a complex interplay between the brain and peripheral metabolic organs. Phylogenetically ancient biogenic monoamine serotonin (5-hydroxytryptamine, 5HT) has an exceptionally important integrative role in regulating energy balance by acting as a key intercellular signaling molecule in both central and peripheral compartments. Due to the inverse relationship between brain 5HT bioavailability and food intake, suggested by early pharmacological studies, 5HT was first recognized as an anorexigenic neurotransmitter [1] and enhancement of 5HT functioning was proposed as a potential anti-obesity treatment (reviewed in [2]). In contrast to central 5HT, the role of peripheral 5HT in the regulation of body weight and energy expenditure/metabolism has only recently been extensively studied [3,4,5,6,7].

The presence of functional 5HT-related proteins (metabolic enzymes, transporters, and receptors) has been demonstrated in various metabolic organs such as pancreas, liver, bone, and adipose tissue [5,8,9,10]. Peripheral 5HT, acting either as a gut-derived hormone or in an autocrine/paracrine manner, has been shown to have important regulatory roles in various metabolic tissues [6,7,11]. Accordingly, 5HT has been shown to stimulate adipogenesis [12,13], regulate lipolysis [14,15], promote insulin secretion [16], inhibit glucose production [14], and blunt brown adipose tissue thermogenic activity [5,17]. Consequently, the role of peripheral 5HT has also been considered in clinically important metabolic disorders such as obesity and type 2 diabetes [10,18]. Based on the discovery that pharmacological inhibition or genetic deletion of a rate-limiting enzyme in peripheral 5HT synthesis protects mice from obesity and insulin resistance [14], reduction of peripheral 5HT signaling has been proposed as a novel strategy to treat obesity [4,7,9,17,18,19].

Due to the extremely complex cooperation between 5HT actions in the brain circuits, the metabolic organs with their own 5HT machinery, and the gut as the main source of circulating 5HT [20], the understanding of the role of 5HT in net energy homeostasis is still quite poor. According to the latest knowledge in the field, increasing 5HT bioavailability in the brain is expected to decrease body weight, whereas increasing peripheral 5HT activity elevates body weight and adiposity [3,6,11,18,21]. However, the relationship between systemic 5HT tone and obesity is not yet entirely clear.

A key regulator of 5HT bioavailability, both in the central and peripheral compartments, is the 5HT transporter (5HTT), a membrane-bound protein that actively removes biologically active 5HT from the extracellular space. Its pharmacological inhibition or genetic deletion leads to a marked increase in extracellular 5HT concentration, resulting in prolonged activation of 5HT receptors [22]. Central and peripheral 5HT compartments are metabolically separated by the blood-brain barrier impermeable for the monoamine [23], but 5HTT in serotonergic neurons and peripheral cells is encoded by the same gene [22]. Therefore, pharmacological or genetic targeting of 5HTT, frequently used to experimentally manipulate 5HT bioavailability, increases 5HT signaling in both the brain and periphery [22].

Increasing whole-body 5HT activity by knocking out the 5HTT gene in rats or mice did not result in a consistent phenotype with respect to energy balance. While reports on glucose intolerance and insulin resistance were quite consistent, findings on body weight and adiposity as well as on blood levels of glucose, insulin, and/or triglycerides in 5HTT knock-out (5HTTKO) animals were rather inconsistent across studies [22,24,25,26,27]. Furthermore, manipulation of 5HT activity by administration of its metabolic precursor or the amine itself showed contradictory effects on circulating glucose/insulin levels [28,29]. In humans, pharmacological inhibition of 5HTT with selective serotonin reuptake inhibitors (SSRIs), which act on both the central and peripheral 5HT systems, has been associated with weight loss, no changes in body weight or mild to moderate weight gain [30,31]. SSRIs are widely used medications for various disorders, so it is of crucial importance to understand how an imbalance in 5HT signaling at the whole-body level affects net metabolic regulation.

In the present study on animals with constitutionally altered serotonergic tone, Wistar-Zagreb 5HT (WZ-5HT) rats [32], we investigated how interindividual differences in endogenous 5HT tone affect energy balance/homeostasis of the organism. The WZ-5HT rat model consists of high-5HT and low-5HT sublines developed by selective breeding of animals toward naturally occurring extremes of platelet serotonin level (PSL) and velocity of platelet serotonin uptake (PSU). As a consequence of genetic selection for platelet 5HT parameters, high-5HT and low-5HT rats have constitutionally different blood 5HT levels, but also show alterations in central 5HT homeostasis. Thus, animals from high-5HT subline not only have higher PSL but also higher biologically active 5HT levels in the platelet-free plasma pool [32], show higher 5HT turnover in the brain and higher KCl-induced elevation in extraneuronal 5HT [32,33], and respond differently to various behavioral challenges [32,33,34,35,36] than low-5HT animals. Therefore, animals from the high-5HT subline are considered to exhibit constitutional hyperactivity of the serotonin system compared to low-5HT rats. Importantly, high-5HT rats exhibit, compared to their low-5HT counterparts, higher body weight and higher food intake throughout life, accompanied by up-regulation of several hypothalamic orexigenic signaling pathways [21]. In addition, high-5HT animals develop some features of the type 2 diabetes phenotype and have decreased bone volume [37].

Metabolic phenotyping of animals with constitutionally different endogenous 5HT tones is continued in the present study, with a particular focus on adipose tissue physiology. Specifically, here we investigated fat accumulation in different depots of white adipose tissue (WAT), metabolic parameters in blood, functional response to glucose and insulin administration, and WAT protein and transcript levels of various body weight-regulating molecules. Overall, the results obtained suggested that increased whole-body 5HT tone is associated with increased adiposity, impaired metabolic homeostasis, and subtle molecular dysregulation of multiple metabolic pathways in white adipose tissue.

## 2. Results

### 2.1. Body Weight and Composition across Lifespan

**Figure 1:**
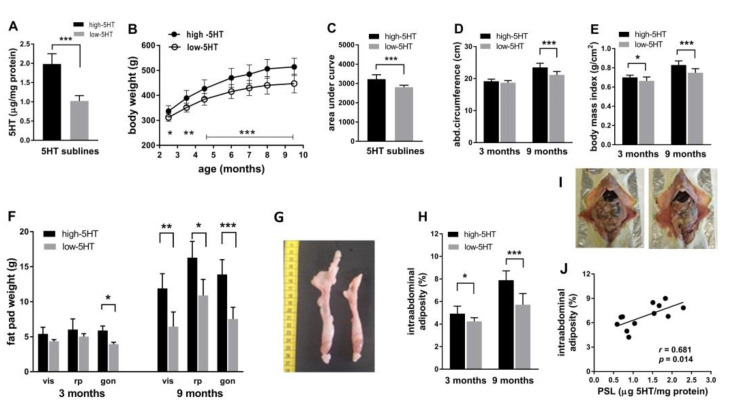
(**A**) Platelet serotonin levels in animals from high-5HT and low-5HT sublines. *n* = 12 rats/subline. Values are representative of all experiments. (**B**) Body weight accumulation in animals from 5HT-sublines measured over 7 months and (**C**) corresponding area under the body weight curve. *n* = 12 rats/subline. (**D**) Abdominal circumference and (**E**) body mass index in 3- and 9-month old male rats from high-5HT and low-5HT sublines. *n* = 12–14 per group. (**F**) Fat mass in visceral (vis), retroperitoneal (rp), and gonadal (gon) adipose tissue depots in 3- and 9-month old males from 5HT-sublines. *n* = 6 per group. (**G**) Gonadal adipose tissue isolated from high-5HT (left) and low-5HT (right) male rats at 9.5 months of age. (**H**) Intra-abdominal adiposity calculated as the sum of visceral, retroperitoneal, and gonadal fat mass and expressed as % of body weight, in 3- and 9.5-month-old male rats from 5HT-sublines. *n* = 6 per group, (**I**) Illustration of visceral cavity of high-5HT (left) and low-5HT (right) males at 9.5 months of age. (**J**) Correlation of platelet serotonin level (PSL) and intra-abdominal adiposity in animals at 9 months of age. *n* = 6 per group, *r* = Pearson correlation coefficient. All: Means ± SD, * *p* < 0.05, ** *p* < 0.01, *** *p* < 0.001.

The differences in platelet serotonin levels (PSL, Figure 1A) and platelet serotonin uptake (PSU) that have arisen between the 5HT-sublines as a result of selective breeding were approximately 2.0-fold. Animals with higher levels of platelet 5HT parameters (PSL and PSU) exhibited a moderately obese phenotype. Small (12%) but significant differences in body weight between 5HT-sublines exist already at birth [21]. Monitoring the body weight of animals from puberty to late middle age showed that high-5HT rats were persistently heavier than low-5HT rats, with magnitudes of differences ranging from 8% at 2.5 months (*p* = 0.045) to 15% at 9.5 months of age (*p* < 0.001) (Figure 1B,C). The differences in abdominal circumference and body mass index between the 5HT-sublines begin to become apparent at 3 months of age and become highly significant by 9.5 months of age (11%, *p* < 0.001, both) (Figure 1D,E). We compared the fat pad weight in various depots of WAT in two age groups of rats from 5HT-sublines. At 3 months of age, high-5HT rats have greater mass of visceral (by 24%), retroperitoneal (by 20%), and gonadal (by 49%, *p* < 0.05) fat compartments and these differences progressively increase with age—at 9.5 months of age, they ranged from 49% in visceral (*p* < 0.05) to 85% in gonadal (*p* < 0.001) fat compartments (Figure 1F). Intra-abdominal adiposity, relative to body weight, was increased in high-5HT subline by 16% (*p* < 0.05) in young animals and by 38% (*p* < 0.001) in mature ones (Figure 1H), and correlated positively with PSL (*r* = 0.681, *p* = 0.014, Figure 1J). Differences in visceral and gonadal adipose tissue between 5HT-sublines are illustrated in Figure 1G,I, respectively.

### 2.2. Blood Metabolic Parameters

**Figure 2:**
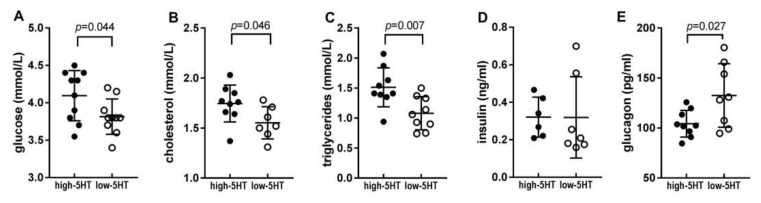
Blood biochemical parameters in fasting male high-5HT and low-5HT rats at 5 months of age. (**A**) Glucose was measured in whole blood, (**B**) total cholesterol and (**C**) triglycerides were measured in serum, and (**D**) insulin and (**E**) glucagon were measured in plasma samples. Data are presented as individual values and means ± SD. *n* = 7–9 per subline, *p*-values are indicated.

Increased adiposity is expected to be associated with changes in circulating metabolic parameters. Our previous study in WZ-5HT females reported higher blood glucose and insulin levels in high-5HT compared to low-5HT animals [37]. In the present study in males, we confirmed the differences between the 5HT-sublines in baseline glucose (Figure 2A), but observed that the presence and magnitude of the differences depended on the length of the period of food deprivation: The longer the food-deprived period, the greater the differences between the 5HT-sublines (Figure S1A). Cholesterol and triglyceride levels were also elevated in high-5HT compared to low-5HT animals, with high-5HT to low-5HT (H/L) ratios being 1.13 (*p* = 0.046) and 1.39 (*p* = 0.007), respectively (Figure 2B,C). Likewise to glucose, triglyceride levels showed a rise in H/L ratio with longer fasting (Figure S1A). No statically significant difference in plasma insulin levels were observed between 5HT-sublines in the present study in males (Figure 2D), while plasma glucagon concentrations were significantly reduced in high-5HT compared to low-5HT animals (H/L = 0.82, *p* = 0.027; Figure 2E).

### 2.3. Adipokine Levels in the Blood

**Figure 3:**
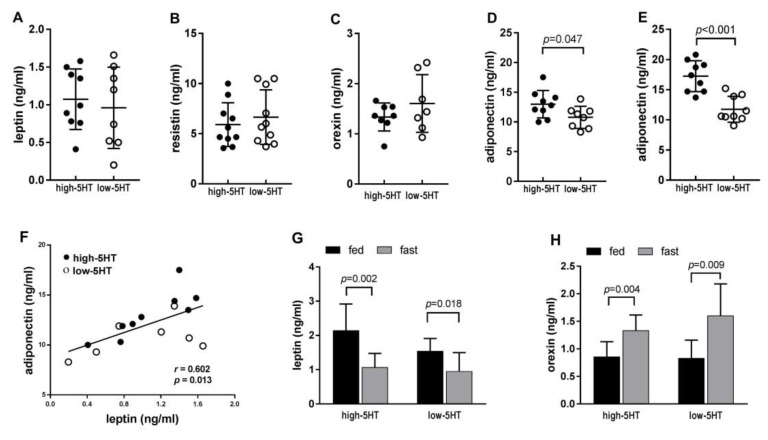
Plasma levels of (**A**) leptin; (**B**) resistin; (**C**) orexin; and (**D**,**E**) adiponectin in overnight fasting male high-5HT and low-5HT rats aged 5 (**A**–**D**) or 9.5 (**E**) months. (**F**) Inter-relation of leptin and adiponectin levels in blood plasma of high-5HT and low-5HT animals. (**G**,**H**) Comparison of plasma leptin (**G**) and orexin (**H**) concentration in fed and fasting animals. Data are presented as individual values and/or means ± SD. *n* = 8–10 per group. *r* = Pearson correlation coefficient, *p*-values are indicated (note: Individual values of plasma levels of leptin and orexin in the fed condition are given in Figure S2).

The metabolic phenotype of the 5HT-sublines was further assessed by evaluating circulating adipokine levels. Contrary to our expectation, there were no statistically significant differences in plasma leptin levels between the 5HT-sublines after overnight fasting (H/L = 1.12, *p* = 0.623, Figure 3A), while in the fed condition, the high-5HT animals had elevated circulating leptin levels (H/L = 1.39, *p* = 0.047, Figure S2A). There were no significant differences between sublines in plasma resistin (H/L = 0.89) or orexin (H/L = 0.83) levels (Figure 3B,C and Figure S2B). Unexpectedly, we observed higher plasma adiponectin levels in obese, high-5HT animals than in lean, low-5HT rats (H/L = 1.21, *p* = 0.047; Figure 3D). This result was replicated in an independent cohort of 9.5-month-old animals and showed even more pronounced differences (H/L = 1.46, *p* < 0.001; Figure 3E). Furthermore, a positive correlation was observed between circulating adiponectin and leptin levels (Figure 3F), being stronger in high-5HT (*r* = 0.836, *p* = 0.005) than in low-5HT (r = 0.501, n.s.) subline. In both 5HT-sublines, fasting induced a reduction in plasma leptin and an increase in plasma orexin levels, as expected. The magnitude of leptin reduction was greater in high-5HT (51%) than in low-5-HT (38%) animals (Figure 3G), while the magnitude of orexin increase was greater in low-5HT (92%) than in high-5HT (55%) animals (Figure 3H). A positive correlation (trend or significant) between plasma adipokine (leptin, adiponectin, orexin) levels and PSL was evident in the low-5HT, but not in the high-5HT subline (Figure S3).

### 2.4. Glucose and Insulin Sensitivity

**Figure 4:**
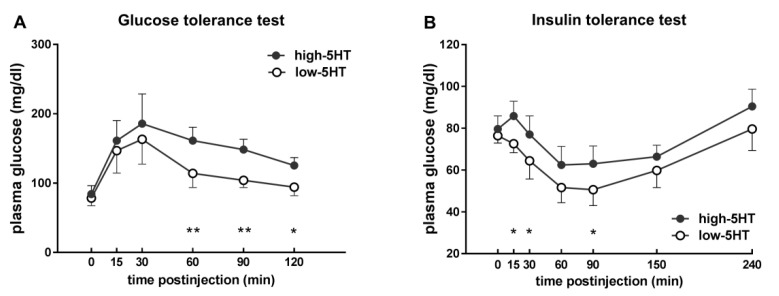
(**A**) Time course of plasma glucose level following glucose administration to 4.5-month-old high-5HT and low-5HT rats subjected to overnight fasting. Means ± SD in groups of 8–9 animals per subline are shown. (**B**) Time course of plasma glucose level following insulin administration in the same groups of animals as in (**A**). Insulin tolerance test was performed after 4 h of fasting and 48 h after the glucose tolerance test. * *p* < 0.05, ** *p* < 0.01.

To assess functional metabolic differences between the 5HT-sublines, we performed a glucose tolerance test (GTT) and an insulin tolerance test (ITT). In 2-month-old animals, there were no significant differences in glucose and insulin tolerance between 5HT-sublines (Figure S4). However, significant differences between the sublines in both GTT (Figure 4A) and ITT (Figure 4B) appeared in older (4.5 months of age) animals, indicating that metabolic differences between sublines progress with age. Specifically, blood glucose levels were higher in high-5HT animals at all time points of GTT, with statistically significant differences between sublines during the return of glucose to corresponding baseline levels (i.e., from 60 min post-injection) (Figure 4A). High-5HT animals also displayed a significantly larger area under the GTT curve (H/L = 1.53, *p* < 0.001). In response to insulin loading in the ITT, high-5HT animals demonstrated a generally smaller decrease in blood glucose levels compared to low-5HT animals (Figure 4B), and an overall smaller area under the ITT curve (H/L = 0.75, n.s.), indicating insulin resistance in high-5HT animals. Of note, most of the high-5HT animals (6 of 9), but only one low-5HT animal, showed a transient increase in glucose levels 15 min after insulin injection, also indicating functional differences between sublines.

### 2.5. Levels of Body Weight-Related Proteins in WAT

**Figure 5:**
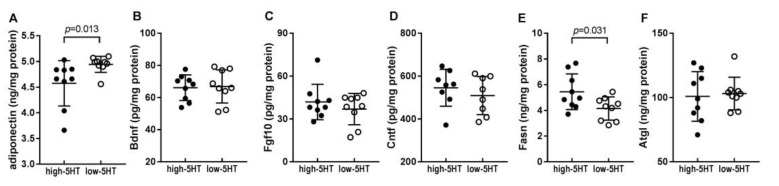
Protein levels of (**A**) adiponectin, (**B**) brain derived neurotrophic factor (Bdnf), (**C**) fibroblast growth factor 10 (Fgf10), (**D**) ciliary neurotrophic factor (Cntf), (**E**) fatty acid synthase (Fasn), and (**F**) adipose triglyceride lipase (Atgl) in the white adipose tissue of high-5HT and low-5HT animals at 4.5 months of age (9.5 months in (**C**)). Data are presented as individual values with means ± SD, *n* = 9–10 per subline, *p*-values are indicated.

We next quantified several body weight-related proteins in WAT of animals from 5HT-sublines (Figure 5). In contrast to plasma levels (Figure 3D,E), WAT levels of adiponectin were significantly reduced in high-5HT compared to low-5HT subline (H/L = 0.92, *p* = 0.013; Figure 5A); similar results were obtained in a cohort of older animals (H/L = 0.90; Figure S5A). Plasma and WAT levels of adiponectin showed a clear trend of negative correlation (Figure S5C). No differences were observed between the 5HT-sublines in WAT protein levels of fibroblast growth factor 10 (H/L = 1.13), brain derived neurotrophic factor (H/L = 1.01), and ciliary neurotrophic factor (H/L = 1.07) (Figure 5B–D).

As to lipid metabolism, the levels of fatty acid synthase were significantly higher in high-5HT animals (H/L = 1.31, *p* = 0.031), while no differences were observed in the levels of the lipolytic enzyme adipose triglyceride lipase (H/L = 1.00) (Figure 5E,F).

### 2.6. Expression Levels of Body Weight-Related Genes in WAT

**Figure 6:**
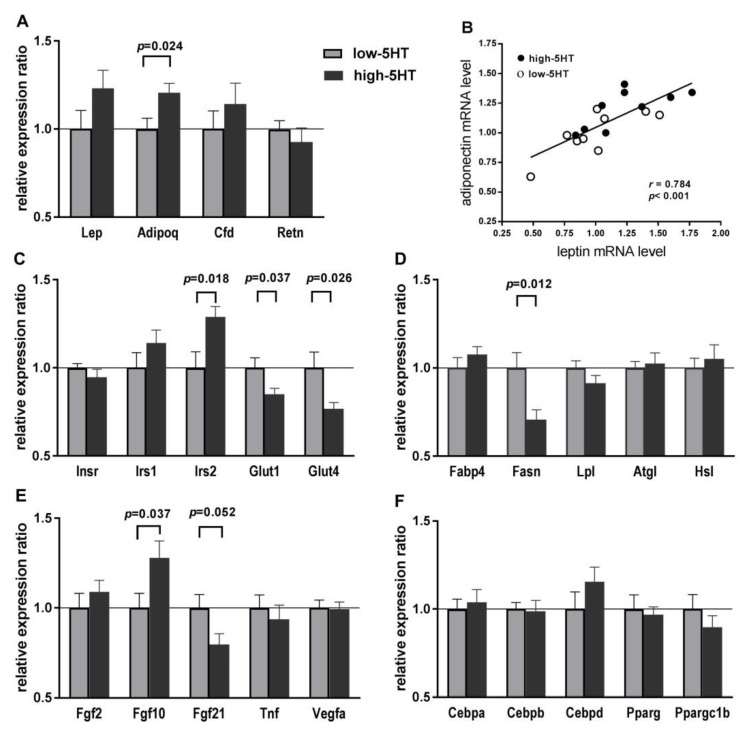
Expression levels of selected body weight-related molecules in white adipose tissue of animals from 5HT-sublines. Relative mRNA levels of (**A**) adipokines, (**C**) carbohydrate-related molecules, (**D**) lipid-related molecules, (**E**) growth factors and (**F**) transcription factors/signaling molecules were measured by qRT-PCR and normalized to the mean of two reference genes. Results are shown as the relative expression ratio between high-5HT and low-5HT sublines (H/L). Means ± SEM are presented, *n* = 9–10. (**B**) Inter-relation of mRNA expression levels of genes encoding leptin and adiponectin in animals from 5HT-sublines (data from (**A**)). *Adipoq*: Adiponectin; *Atgl*: Adipose triglyceride lipase; *Cebp*: CCAAT/enhancer binding protein; *Cfd*: Complement factor D (adipsin); *Cntf*: Ciliary neurotrophic factor; *Fabp4*: Fatty acid binding protein 4; *Fasn*: Fatty acid synthase; *Fgf*: Fibroblast growth factor; *Glut*: Glucose transporter; *Hsl* (*Lipe*): Hormone sensitive lipase; *Insr*: Insulin receptor; *Irs*: Insulin receptor substrate; *Lep*: Leptin; *Lpl*: Lipoprotein lipase; *Pparg*: Peroxisome proliferator-activated receptor gamma; *Ppargc1b*: Parg-gamma coactivator 1 beta; *Retn*: Resistin; *Tnf*: Tumor necrosis factor; *Vegfa*: Vascular endothelial growth factor A.

To further understand which molecules/pathways contribute to the functional differences between 5HT-sublines, we analyzed WAT expression levels of various classes of body weight-related genes. As shown in Figure 6, adiponectin mRNA levels in WAT were upregulated in high-5HT compared to low-5HT animals (H/L = 1.21, *p* = 0.024), while differences in mRNA levels of leptin (H/L = 1.23) and complement factor D/adipsin (H/L = 1.16), although similar in magnitude, did not reach statistical significance (Figure 6A). There was a positive correlation between the expression levels of leptin and adiponectin (Figure 6B) in both high-5HT (*r* = 0.699, *p* = 0.035) and low-5HT (*r* = 0.7927, *p* = 0.010) sublines. Among the genes involved in glucose homeostasis, insulin receptor substrate 2 (*Irs2*) mRNA was upregulated (H/L = 1.29, *p* = 0.018), while glucose transporter 1 (*Glut1*) (H/L = 0.85, *p* = 0.037) and glucose transporter 4 (*Glut4*) (H/L = 0.77, *p* = 0.026) mRNAs were downregulated in high-5HT compared to low-5HT animals (Figure 6C). Among the genes involved in lipid metabolism, the expression of fatty acid synthase (*Fasn*) was significantly reduced in the high-5HT subline (H/L = 0.71, *p* = 0.012), while other analyzed genes showed no differences between sublines (Figure 6D). Among the genes encoding growth factors, fibroblast growth factor 10 (*Fgf10*) expression was upregulated (H/L = 1.29, *p* = 0.018), while fibroblast growth factor 21 (*Fgf21*) expression was downregulated (H/L = 0.79, *p* = 0.052) in high-5HT compared to low-5HT sublines (Figure 6E). No significant differences in the WAT expression levels of several genes encoding transcriptional/regulatory factors involved in energy expenditure were observed between the 5HT-sublines (Figure 6F).

### 2.7. Adipogenesis and Obesity PCR Arrays

Due to the rather small molecular differences observed between the 5HT-sublines in adipose tissue, we decided to compare their gene expression profile on a larger scale using the PCR array technology and RNA pools from six mature (10-month-old) animals per subline. The unprocessed results of the Rat Adipogenesis and Rat Obesity RT2 Profiler PCR Arrays are shown in Tables S1 and S2, respectively. Of the total 160 genes examined, 104 showed significant expression in the WAT using a quantification cycle (Cq) of less than 30 as a criterion. Several genes that overlapped between the two arrays (*Adipoq*, *Insr*, *Lep*, *Ppara*, *Pparg*, *Ppargc1a*) showed well-matched expression level results.

**Figure 7:**
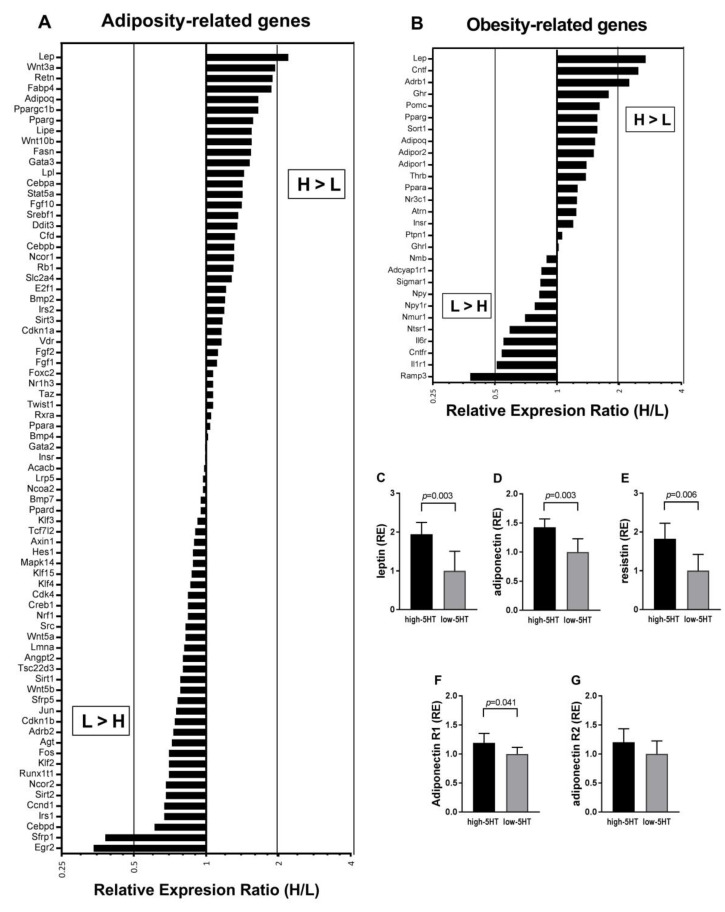
Gene expression levels in white adipose tissue (WAT) of high-5HT (H) and low-5HT (L) animals at 10 months of age. (**A**,**B**) Genes with a quantification cycle (C_q_) of less than 30 detected in visceral WAT using (**A**) rat adipogenesis and (**B**) rat obesity RT^2^ profiler PCR arrays. Results are presented as the relative expression ratio (RER) between the high-5HT and low-5HT subline (H/L), and genes are sorted in descending order. Gene names are listed in Supplementary Tables S1 and S2. (**C**–**G**) Relative expression (RE) of adipokines and their receptors analyzed in individual animals by qRT-PCR analysis: (**C**) leptin, (**D**) adiponectin, (**E**) resistin, (**F**) adiponectin receptor 1, and (**G**) adiponectin receptor 2. Gene expression levels were normalized to the mean of five reference genes (**A**,**B**) or to actin-beta (**C**–**G**) as the reference gene. Histograms in **C**–**G** represent means ± SD, *n* = 6.

In general, the differences in gene expression levels between 5HT-sublines were rather small. Among 104 genes, only eight genes were with more than 2.0-fold change and a total of 34 genes were with more than 1.5-fold change (Figure 7A,B). Several genes were expressed only in the low-5HT subline (Cq < 30), so they were excluded from the data presented in Figure 7, where the array results are shown as the ratio of high-5HT to low-5HT subline (H/L). Genes that were upregulated in high-5HT compared to low-5HT animals could be tentatively classified into several categories, such as (1) adipokines (*Lep*, *Retn*, *Cfd*, *Adipoq*) and their receptors (*Adipor1*, *Adipor2*); (2) enzymes and other proteins involved in lipid metabolism (*Fasn*, *Lipe*, *Lpl*, *Fabp4*); (3) growth factors (*Cntf*, *Fgf10*), and (4) regulatory/signaling molecules (*Sort1*, *Srebf1*, *Pparg*, *Ppargc1b*, *Cebpa*, *Cebpb*, *Stat5a*, *Gata3*, *Ncor1*, *Wnt10b* and *Wnt3a*). In addition, increased expression levels of some (5) hormone and neurotransmitter receptors (*Ghr*, *Adrb1*, *Thrb*) were detected in the high-5HT subline. Genes that were downregulated in high-5HT compared to low-5HT animals included (1) regulatory/signaling molecules (*Fos*, *Jun*, *Klf2*, *Sfrp1*, *Runx1t1*, *Ncor2*, *Cebpd*); (2) cell cycle regulators (*Ccnd1*, *Cdkn1b*); (3) intracellular transporter proteins (*Sirt1*, *Sirt2*, *Ramp3*), and (4) receptors for growth factors (*Cntfr*), citokines (*Il1r1*, *Il6r*), and neurotransmitters (*Adrb2*, *Ntsr1*, *Nmur1*).

To assess the accuracy of the array gene expression results, we performed validation of the array data by qRT-PCR analysis in individual samples. For validation, we selected five genes that were upregulated in high-5HT animals according to the array results. The results of qRT-PCR analysis confirmed that the mRNA levels of four of these genes were significantly higher in high-5HT animals compared with low-5HT animals, and one of these genes (*Adipor2*) showed a nonsignificant trend in the same direction as in the array data (Figure 7C–G).

## 3. Discussion

Previous studies on the WZ-5HT rat model show that the increased whole-body 5HT tone promotes the development of a moderate obesity phenotype in adulthood. We have reported differences in brain hypothalamic mechanisms between the 5HT-sublines, specifically upregulation of orexigenic signaling peptides and increased feeding behavior in animals with high 5HT tone compared to animals with low 5HT tone [21]. It is important to note that food intake in high-5HT compared to low-5HT rats was increased when expressed per animal but decreased when adjusted for body weight, suggesting that other mechanisms besides feeding behavior contribute to the obese phenotype of high-5HT rats. Indeed, it has been suggested that an increase in brain 5HT bioavailability induces body weight loss, while an increase in peripheral 5HT bioavailability leads to weight gain [3,5,6,11]. In our sublines, 5HT signaling is affected at both brain and peripheral levels [32], which on the one hand is a drawback of the model, as it is not possible to distinguish between central and peripheral effects, but on the other hand, may be an important advantage as it may better reflect natural situations [21]. The 2.0-fold difference in platelet 5HTT activity between 5HT-sublines is similar to the degree of variation in 5HTT expression levels in the healthy human population [22,38], so our model may well mimic the physiological situation in humans. In the present work, we report on metabolic differences between 5HT-sublines that are triggered by their different endogenous 5HT tone. The focus is on adipose tissue as one of the metabolic organs coordinating whole-body energy homeostasis [39], but the WZ-5HT model could be useful to study the impact of individual 5HT tone on the crosstalk between various metabolic organs in obesity.

Intra-abdominal adiposity, expressed relative to body weight, differs by almost 40% between the mature animals from 5HT-sublines (Figure 1H). Adiposity generally results from an imbalance between energy intake and expenditure, and 5HT signaling modulates both processes. Differences in body fat between animals from 5HT-sublines are probably not due to differences in locomotor activity [34,35] but could be due to differences in food intake [21] and/or brown adipose tissue thermogenesis [21,40]. The increased adiposity of the high-5HT rats is compatible with the reported obesity of 5HTTKO mice [22,24] which, similarly to our high-5HT rats [32], have increased extracellular 5HT pools in the brain and periphery. The finding is also in line with a part of clinical studies showing an association of long-term SSRI use with an increased risk of developing obesity [30,31]. However, there is no consensus in the literature regarding circulating serotonin levels in obesity, with studies reporting both positive [41,42,43,44] and negative [45,46,47,48] or no [48,49] associations between blood 5HT parameters and body mass index/body fat. The relationship between circulating 5HT levels and obesity is mostly studied by measuring the platelet 5HT pool, while data on biologically active 5HT in blood plasma and activity of platelet 5HT transporter are scarce. Our data link higher 5HT levels in both blood 5HT pools as well as higher platelet 5HT transporter activity [32] to increased body fat accumulation.

In accordance with the well-established link between abnormal fat deposition and glucose dysregulation [50], our high-5HT animals exhibit hyperglycemia (Figure 2A) and impaired glucose tolerance and insulin sensitivity (Figure 4), both of which progress with aging. These functional changes are associated with upregulated *Irs2*, but downregulated *Glut4* and *Glut1* expression in WAT (Figure 6C), indicating that both signaling downstream of the insulin receptor as well as insulin-dependent and -independent glucose uptake in adipocytes contribute to insulin resistance in high-5HT animals. The functional metabolic divergence between the 5HT-sublines is also reflected in the observation that the differences between them in blood glucose and lipid levels become greater with longer periods of fasting (Figure S1A). This may be related to a direct 5HT effect on glucose metabolism, as 5HT has been shown to promote liver gluconeogenesis, inhibit glucose uptake, and induce lipolysis during fasting [14].

In addition to adipose tissue, dysregulation in functioning of other metabolic tissues likely contributes to impaired glucose homeostasis in high-5HT animals. Previously, we have shown increased number and size of pancreatic beta-cell islets and decreased bone volume in high-5HT animals [37]. Here, we showed diminished circulating glucagon levels in high-5HT rats (Figure 2E), which is a further evidence of pancreatic dysregulation. Stern et al. [51] have reported that obesity dysregulates fasting-induced changes in circulating glucagon and the downstream effects of its signaling at the liver [51]. In their study in mice, fasting increased glucagon secretion in lean but not obese animals, and similar results have been shown in humans. These findings are consistent with our results showing higher fasting plasma glucagon levels in lean (low-5HT) than in obese (high-5HT) rats. This could be due to enhanced somatostatin-mediated inhibition of glucagon secretion in obese, high-5HT rats, as was the case in the study by Stern et al. Alternatively or in addition, the decreased fasting plasma glucagon levels in this subline could be a consequence of enhanced inhibition of glucagon secretion by paracrine 5HT signaling in the pancreas, as shown [52].

Overall, dysregulation of WAT and the pancreas, but probably other metabolic tissues as well, likely contributes to metabolic deficits in high-5HT animals.

Metabolic homeostasis in relation to 5HT system functioning has been studied in various animal models. For example, pharmacological inhibition of peripheral 5HT synthesis in animals on a high-fat diet leads to improved glucose tolerance and insulin sensitivity [5,17]. Furthermore, 5HTT deficiency induces adiposity and insulin resistance that occurs before the increase in adiposity, suggesting that it is not a mere consequence of obesity [26]. In humans, a low-expressing allele of the 5HTT gene polymorphism is associated with poor glucose control [53], while chronic pharmacological inhibition of 5HTT often leads to weight gain/obesity [30,31]. Although based on different manipulations, all these models share increased 5HT bioavailability, which is also present in our high-5HT subline. Therefore, the association between increased 5HT availability and metabolic deficit is also confirmed by our model.

Regarding lipid metabolism, high-5HT compared to low-5HT rats showed increased adipose tissue protein levels of a major lipid-synthesizing enzyme Fasn (Figure 5E), suggesting increased lipogenesis in the WAT of high-5HT animals. This finding is consistent with the increasing effect of 5HT on *de novo* lipogenesis leading to obesity-prone adipocytes [13]. The effect of 5HT on lipogenesis is mediated through the 5HT2A receptor [13], and our high-5HT animals have increased 5HT2A receptor mRNA and protein levels in WAT (preliminary results). It should be noted that the differences in Fasn protein levels between the sublines were only partially accompanied by analogous changes in *Fasn* mRNA levels, i.e., in one (Figure 7A and Table S1, mature rats) but not in another (Figure 6D, adult rats) group of animals. Similarly, previous studies reported either decreased [54,55] or increased [56,57] *Fasn* expression levels in adipose tissue from obese humans and animal models of obesity. Collectively, these data suggest complex *Fasn* gene regulatory mechanisms operating at both transcriptional and translational levels. In contrast to Fasn, protein levels of the lipid-catabolizing enzyme Atgl (Figure 5F) as well as mRNA levels of triglyceride breakdown enzymes (*Atgl*, *Hsl*) were similar in the WAT of high-5HT and low-5HT animals. The effect of 5HT on lipolysis has been described as either inhibitory [15] or stimulatory [14], whereas our results suggest no changes in WAT expression levels of lipolytic enzymes in response to differential endogenous 5HT activity. However, it is possible that lipolysis differs between our sublines due to the altered lipolytic enzyme activity, which we did not directly investigate here. Findings that high-5HT animals have decreased levels of glucagon, which activates lipolysis via transcription-independent mechanisms [58], argue for decreased lipolytic activity in this subline, at least in response to fasting. Since glucagon primarily acts on the liver, possible differences between sublines in the expression/activity of lipolytic enzymes could be more pronounced in this organ.

Transcriptional profiling of WAT showed that several genes involved in lipid metabolism, particularly fatty acid metabolism, were among the most upregulated genes in high-5HT animals (Figure 7 and Tables S1 and S2). The absence of significant differences between 5HT-sublines in mRNA levels of genes responsible for adipocyte differentiation, such as *Pparg* [59], indicate that in younger animals (Figure 6) adipocyte development may not have a major contribution to differences in fat expansion between 5HT-sublines.

In contrast to blood glucose, cholesterol, and triglyceride levels (Figure 2A–C), circulating adipokine (leptin, resistin, orexin) levels (Figure 3A–C) did not differ between 5HT-sublines, suggesting that adult obese high-5HT rats are not leptin resistant. However, a comparison of plasma adipokine levels between fed and fasting states (Figure 3G,H) revealed a more pronounced response to fasting (higher increase in leptin and decrease in orexin levels) in the low-5HT animals, corroborating the functional metabolic differences between the 5HT sublines.

The upregulation of mRNA levels of adipokines in WAT of high-5HT rats, observed in both adult and mature animals, is consistent with their increased adiposity and the role of 5HT in promoting adipogenesis. Intriguingly, both plasma adiponectin levels and its transcript levels in the WAT were increased in high-5HT rats, which contradicted our expectations based on the increased adiposity of these animals. In general, circulating adiponectin levels are reduced in rodent and human obesity [20], although there are few studies consistent with our findings. For example, plasma adiponectin levels were elevated in female 5HTTKO mice which, similarly to our high-5HT rats, exhibit an obese phenotype [25], and were decreased in animals with pharmacologically inhibited 5HT synthesis, which exhibit reduced fat mass [5]. Authors of the latter study [5] concluded that the decrease in circulating adiponectin levels was due to the reduced fat mass and not to the downregulation of its adipocyte expression by inhibition of 5HT synthesis. However, a possible regulatory role of 5HT in adiponectin production/release from adipocytes should also be considered. It has been reported that the genetic or pharmacological inhibition of the 5HT2A receptor leads to increased adiponectin expression in differentiated adipocytes [60]. As previously mentioned, our high-5HT animals show increased 5HT2A receptor levels in WAT, supporting a role of 5HT signaling in the regulation of adiponectin expression.

Adiponectin protein levels in WAT were lower in high-5HT compared to low-5HT animals, thus in contrast to WAT mRNA levels as well as plasma protein levels. We have no explanation for this divergence, but it should be noted that hypertrophic rather than hyperplastic adipose tissue expansion is characterized by decreased adiponectin in adipocytes, defective Glut4 trafficking, and impaired insulin sensitivity [61], all hallmarks of our high-5HT animals. This might suggest that fat expansion in high-5HT animals is primarily hypertrophic in origin. In recent years, adiponectin has emerged as an important regulator of energy homeostasis with an extremely complex regulation and interactions with other regulatory molecules [62]. Our data, including those indicating a correlation between platelet 5HT and adiponectin levels only in low-5HT animals (Figure S3), add to this complexity. It is worth noting that plasma levels of adiponectin and leptin show a significant positive correlation, and similar was observed for their WAT mRNA levels.

Among molecules known to be physiological regulators of body weight/energy homeostasis are several members of the fibroblast growth factor (Fgf) family, with Fgf21 recognized as a key hormone with beneficial effects on insulin sensitivity and glucose/lipid metabolism [63,64]. Fgf21 stimulates glucose uptake in adipocytes via induction of Glut1 expression/activity [65]. Both *Fgf21* and *Glut1* mRNAs were downregulated in high-5HT compared to low-5HT animals, indicating that their expression in adipocytes is regulated by the endogenous 5HT tone. It has been recently reported that the decrease in hepatic *Fgf21* expression in obese mice is mediated through 5HT2A and 5HT2B receptors [66] and, based on differential expression of 5HT2A receptors in the WAT of our sublines, we assume that something similar may also occur in adipose tissue. Fgf21 shares many functional similarities with adiponectin [67]. Both hormones improve insulin sensitivity and glycemic control, and ameliorate dyslipidemia [68]. In white adipocytes adiponectin acts as a downstream effector of Fgf21 and mediates the systemic effects of Fgf21 on energy metabolism and insulin sensitivity [69]. Here, we found decreased WAT adiponectin protein and *Fgf21* mRNA levels in metabolically compromised high-5HT animals compared to low-5HT animals. The functional connection of Fgf21 and adiponectin has been suggested to have implications for their therapeutic potential [67,68] and our results indicate that both hormones may be regulated by the endogenous 5HT activity.

In contrast to Fgf21, WAT mRNA levels of Fgf10, which is known to be a stimulator of adipogenesis [67], were upregulated in obese high-5HT animals. An identical change in *Fgf10* transcript levels were found in hypothalami [21], however in both WAT and hypothalami an increase in *Fgf10* mRNA levels was not accompanied by changes at the protein level, indicating a complex control of its level. Fgf10 has also been proposed as a candidate for novel anti-obesity drugs [67,70] and our rat model may be useful to investigate its potential therapeutic efficacy. As for the other analyzed growth factors associated with obesity (Fgf2, Bdnf, Cntf, Tnfα, Vegf), we found no evidence of their differential WAT expression between 5HT-sublines, either at the protein or mRNA level. However, it should be noted that PCR array experiments showed upregulation and downregulation of *Cntf* and its receptor mRNA, respectively, in mature high-5HT animals, indicating that the differences between 5HT-sublines become more pronounced with aging. In contrast to WAT, hypothalamic *Cntfr* mRNA levels were upregulated in the high-5HT subline [21], indicating the opposing regulation of this neurotrophin by central and peripheral 5HT.

By PCR array experiments, we gained comprehensive insight into WAT molecular pathways that may be affected by lifelong alterations in 5HT homeostasis in mature (10-months-old) animals. Since the 5HTT activity in 5HT-sublines is still within the range of physiological values [32], only limited changes in expression levels were expected, as indeed shown also by qPCR experiments. Therefore, we focused on gene groups rather than individual genes when interpreting PCR array results, and adopted relatively low stringency for the comparison of 5HT-sublines. In addition to the upregulation of mRNAs for various adipokines, shown also by qPCR experiments, WAT expression profiling by PCR arrays revealed upregulation of genes involved in lipid metabolism (*Fasn, Fabp4, Lipe, Lpl*) and lipogenic transcription factors (*Cebpa, Pparg, Ppargc1b, Srebf1*) in high-5HT rats, findings not observed in qPCR experiments in younger animals. Pparg is a master regulator of adipogenesis and 5HT has been shown to be its high-affinity agonist [71], so 5HT would be expected to act as a direct lipogenic stimulus in WAT via regulation of transcription factors, as shown in liver [72]. Members of the JAK/STAT signaling cascade involved in cell differentiation (*Stat5a, Cebpb, Gata3, Ghr*) also appear to be upregulated in the high-5HT subline. On the other hand, the anti-adipogenic signaling cascade triggered by Wnt (*Sfrp1, Wnt5a, Wnt5b, Ccnd1*) as well as some members of the Mapk signaling pathway (*Jun, Fos*) were downregulated in the high-5HT rats. Collectively, according to the PCR array results, transcription factors regulating the first stages of adipocyte differentiation [73] were downregulated in high-5HT animals, whereas those regulating the final steps of adipocyte maturation [73] were upregulated. WAT of high-5HT rats also showed altered (up or downregulated) mRNA expression levels of various receptors, indicating a differential functional response of 5HT-sublines to afferent signals from the periphery and brain.

In conclusion, this study provides evidence that the constitutionally increased 5HT tone is associated with adiposity and impaired/dysregulated metabolic homeostasis. Our results in the genetic rat model confirm some known and suggest some novel interrelations between the 5HT activity and metabolic functioning. In particular, the possibility that actions of adiponectin and Fgf21, both of which have therapeutic potential against obesity, under the physiological control of endogenous 5HT tone may be important from a clinical perspective. In addition, our WZ-5HT rats could be a useful animal model to study the integrative serotonergic mechanisms in dysmetabolic conditions such as obesity and insulin resistance.

## 4. Materials and Methods

### 4.1. Animals

Studies were performed on two sublines of Wistar-Zagreb 5HT (WZ-5HT) rats obtained by selective breeding for the extreme values of platelet serotonin level (PSL) and platelet serotonin uptake (PSU) at Ruđer Bošković Institute (Zagreb, Croatia). The generation of 5HT-sublines has been described previously [32,74,75]. Just in brief, males and females with the highest and the lowest values of platelet 5HT parameters were mated to generate high-5HT and low-5HT sublines, respectively. Determinations of PSL and PSU were performed in offspring of each generation and animals displaying the extreme values were selected as parents for the next generation. The divergence of mean platelet 5HT levels stabilizes at approximately 70% (low-5HT subline) and 150% (high-5HT subline) of the mean value of the initial population. Selective breeding was restarted several times over the years with similar dynamics of divergence and a final range of differences between sublines. In this study, young (2–3 months), adult (4.5–6 months), and mature (9–9.5 months) male animals were used (age specified in figure legends). Animals were housed three per cage under controlled conditions (temperature 22 ± 2 °C; humidity 55 ± 10%, and 12 h light-dark cycle) with standard rat chow (4RF21, 3.9 kcal/g, 6.55% kcal from fat, Mucedola, Italy) and water available ad libitum.

All experiments were approved by the institutional and national (Ministry of Agriculture, Republic of Croatia) ethical committees and were conducted in accordance with the ILAR Guide for the Care and Use of Laboratory Animals and Croatian animal protection law (NN 135/06 and 37/13).

### 4.2. Body Weight Measurements and Phenotyping

Body weight accumulation was monitored in groups of 12 animals per 5HT subline at regular intervals from puberty to 9.5 months of age. Degrees of overall obesity of animals were assessed in another cohort of 5HT-sublines aged 3 (*n* = 6) and 9 months (*n* = 6), by determination of abdominal circumference (measured as diameter of the abdominal region at the largest point of the abdomen) and body length (measured from the tip of the nose to the base of the tail using a millimeter paper) of isoflurane anesthetized animals and calculation of their body mass index (BMI, ratio of body weight, and body length^2^).

### 4.3. Tissue/Sample Collection

Rats were euthanized and their visceral, retroperitoneal, and/or gonadal white adipose tissue (WAT) depots were manually dissected and weighted. Intra-abdominal adiposity (IAA) was calculated as the ratio of the total intra-abdominal fat mass (calculated as a sum of isolated fat depots) and total body mass. For gene/protein expression analyses, small portions of WAT were rapidly removed and either immediately frozen in liquid nitrogen for array expression analyses (cca 90 mg) and ELISA determination (200–300 mg) or placed in a RNAlater solution as recommended by the manufacturer (Qiagen, Germantown, MD, USA) for RT-qPCR analyses (cca 60 mg). All samples were stored at −80 °C until further analyses.

### 4.4. Determination of Platelet 5HT Parameters

PSL was determined spectrophotometrically and the velocity of PSU was measured radiochemically in the same blood sample obtained from rat jugular vein under isoflurane anesthesia (SomnoSuite anesthesia system, Kent Scientific, Torrington, CT, USA) as described previously [75,76].

### 4.5. Biochemical Measurements

Blood for obtaining plasma and serum samples was collected from the jugular vein into tubes containing EDTA (BD Vacutainer™ K2E) or gel activator (BD Microtainer^®^ SST II), respectively. Plasma samples were centrifuged (1000× *g*, 10 min), aliquoted, and stored at −80 °C until analyses. Enzyme-linked immunosorbent assays (ELISA) were used to determine leptin, adiponectin, insulin (Demeditec Diagnostics, Kiel, Germany), glucagon, resistin, and orexin (ElabScience, Wuhan, China) plasma levels according to the protocol provided by the manufacturers. The assay sensitivities were 10 pg/mL (leptin), 0.081 ng/mL (adiponectin), 0.190 ng/mL (resistin), 0.1 ng/mL (insulin), and 37.5 pg/mL (glucagon, orexin). Serum samples were left to clot before centrifugation (1000× *g*, 15 min) and total cholesterol, high-density lipoprotein (HDL) cholesterol, and triglycerides were measured automatically using the clinical chemistry analyser (Olympus AU480, Beckman Coulter, Brea, CA, USA).

The blood glucose level was determined in blood samples taken from a cut at the tip of the tail by the glucose-oxidase method using a reflectance glucometer (Super Glucocard II, Arkray, Kyotp-fu, Japan). Blood parameters were determined in animals that were either in fed state or subjected to fasting for a period of 4, 12 or 18 h (specified in the figure legends).

For measuring selected proteins’ levels, adipose tissue was homogenized in 1:5 *w/v* tissue protein extraction reagent (T-PER, Thermo Scientific, Waltham, MA, USA) with protease inhibitor (Halt Protease Inhibitor Cocktail, Thermo Scientific, Waltham, MA, USA) added. The resulting lysates were centrifuged (10,000× *g*, 10 min) and the supernatants were assayed for adiponectin, brain derived neurotrophic factor (Bdnf), ciliary neurotrophic factor (Cntf), fibroblast growth factor 10 (Fgf10), fatty acid synthase (Fasn), and adipose triglyceride lipase (Atgl) levels according to the manufacturers’ (Elabscience, Wuhan, China) protocols. Assay sensitivities were 0.081 ng/mL for adiponectin, 18.75 pg/mL for Bdnf, 7.50 pg/mL for Cntf, 9.38 pg/mL for Fgf10, 0.19 ng/mL for Fasn, and 0.47 ng/mL for Atgl. The total protein content in homogenates was determined by the Bradford method.

### 4.6. Metabolic Phenotyping

The glucose tolerance was assessed by the glucose tolerance test (GTT). After 12 h of fasting, the baseline blood glucose was measured in blood samples taken from the tail vein. Subsequently, a 20% glucose solution was administered by intraperitoneal (i.p.) injection at a concentration of 2 g/kg body weight, and the glucose level was measured in blood collected via the tail bleed at 15, 30, 60, 90, 120 min post-injection using a glucometer. A single cut at the tip of the tail was enough to collect all of the blood samples.

Peripheral insulin sensitivity was assessed by the insulin tolerance test (ITT), which was carried out 48 h after the GTT. The baseline glucose level was determined in 4 h fasted animals followed by i.p. administration of insulin (Humulin R, Eli Lilly, Indianopolis, IN, USA) at a concentration of 0.5 unit/kg body weight and monitoring of blood glucose level at 15, 30, 60, 90, 150, and 240 min thereafter.

### 4.7. Gene Expression Analyses

Total RNA was isolated from WAT (approximately 90 mg per sample) using the RNeasy Lipid Tissue Kit (Qiagen, Germantown, MD, USA) according to the manufacturers’ protocol including the optional on-column DNA digestion step. The concentration and purity of isolated RNAs were assessed by spectrophotometry (NanoDrop, ND-1000, Thermo Fisher Scientific, Waltham, MA, USA). Aliquots of RNA were run on 1% agarose gel electrophoresis to verify the integrity. All the samples showed sharp 28S and 18S bands at a ratio of approximately 2:1. RNA samples were stored at −80 °C until further processing.

The relative levels of specific mRNAs were determined by reverse transcription (RT)—quantitative PCR (qPCR) based on SybrGreen detection chemistry. The cDNA was synthesized from equal amounts of RNA per sample, using High Capacity RNA to cDNA Synthesis Kit (Applied Biosystems, Thermo Fisher Scientific, Waltham, MA, USA) according to the manufacturers’ protocol. The cDNA from the pool of all RNA samples was synthesized to be used for preparing standard curves. Control reactions without reverse transcriptase (no-RT) were prepared to test for contamination with genomic DNA. The cDNA samples were stored in small aliquots at −20 °C. Sequences of the primers used in qPCR are listed in Table S3. The qPCR assays were prepared using the Fast SYBR Green Master Mix and were run on a StepOnePlus Real-Time PCR System (both from Applied Biosystems Thermo Fisher Scientific, Waltham, MA, USA) according to the manufacturers’ protocols. Each qPCR plate contained a 5-point standard curve with 2.0-fold serial dilutions and all the reactions were performed in duplicates at a minimum. The specificity of amplicons was verified by the agarose gel electrophoresis and melting curve analysis. No-RT controls yielded undefined C_q_ or C_q_ more than 15 cycles higher than those of the respective cDNA samples. Relative expression levels determined by a relative standard curve method [77] were normalized to the mean of two reference genes, glyceraldehyde-3-phosphate dehydrogenase (*Gapdh*) and actin beta (*Actb*).

Rat adipogenesis and rat obesity RT^2^ profiler PCR arrays (Qiagen, Germantown, MD, USA) were used to measure the expression levels of 160 genes involved in the regulation of adipogenesis/obesity (see www.qiagen.com for a complete list of genes, accessed on 21 May 2012). First strand cDNAs were synthesized from RNA pools obtained from six animals per subline, using the RT^2^ First Strand Kit (Qiagen, Germantown, MD, USA) according to the manufacturers’ instructions. PCR array assays were prepared using the RT^2^ qPCR SYBR Green/ROX MasterMix (Qiagen, Germantown, MD, USA) according to the manufacturers’ recommendations. Reactions were performed on a 7300 Real Time PCR System (Applied Biosystems Thermo Fisher Scientific, Waltham, MA, USA) using standard cycling conditions followed by the dissociation curve analysis. The positive PCR control for inter-assay comparison and the controls for genomic DNA contamination and reverse transcription all passed quality control. Genes with a quantification cycle (C_q_) greater than 30 were considered to have very low or no expression and were excluded from further analysis. The analyzed genes are listed in Tables S1 and S2. The expression of each gene of interest was normalized to the mean of five housekeeping genes (*Rplp1, Hprt1, Rpl13a, Ldha,* and *Actb*). The fold change in relative expression level in the high-5HT as compared to low-5HT subline was calculated using the comparative C_q_ (ΔΔC_q_) method [78].

### 4.8. Statistical Analysis

Statistical analyses were performed using GraphPad Prism, v 7.05 (GraphPad Software, San Diego, CA, USA) Normality of data distribution was tested by the D’Agostino-Pearson omnibus test, homogeneity of variances by Bartlett’s test, and presence of outliers by the Grubbs test. The means between two groups were compared with the two-tailed unpaired Student’s t-test (with Welch’s correction if variances were significantly different) or Mann-Whitney U-test, as appropriate. Outliers were excluded from the analysis. The total area under the curve (AUC) was calculated using the linear trapezoidal method. The relationship between parameters was evaluated by the Pearson correlation coefficients. Time-monitored parameters were analyzed by the repeated measures analysis of variance (RM-ANOVA) followed by Fisher’s LSD test and using independent samples t-tests. Results are presented as individual values and/or group means (M) with standard deviation (SD) or standard error of the mean (SEM). Differences were considered statistically significant if *p* < 0.05.

## Data Availability

All data generated for this study are included in the article/Supplementary Material.

## Supplementary Materials

The following are available online at https://www.mdpi.com/article/10.3390/ijms22105400/s1, Figure S1: Ratio of blood metabolic parameters between high-5HT and low-5HT subline in relation to feeding status; Figure S2: Plasma levels of leptin and orexin in fed animals; Figure S3: Relationship between platelet serotonin level and plasma levels of adipokines; Figure S4: Glucose and insulin tolerance tests in young animals from 5HT-sublines; Figure S5: Adiponectin level in white adipose tissue of high-5HT and low-5HT animals; Table S1: List of genes analysed in white adipose tissue using the Rat Adipogenesis RT^2^ Profiler PCR Array; Table S2: List of genes analysed in white adipose tissue using the Rat Obesity RT^2^ Profiler PCR Array; Table S3: Primer sequences used in RT-qPCR analysis.

## Abbreviations

*Actb*, actin beta; *Adipoq*, adiponectin; *Adipor,* adiponectin receptor; *Adrb*, beta adrenergic receptor; *Atgl*, adipose triglyceride lipase; *Bdnf,* brain derived neurotrophic factor; *Ccnd1*, cyclin D1; *Cdkn1b*, cyclin-dependent kinase inhibitor 1A; *Cebp*, CCAAT/enhancer binding protein; *Cfd*, complement factor D (adipsin); *Cntf*, ciliary neurotrophic factor; *Cntfr*, ciliary neurotrophic factor receptor; *Fabp4*, fatty acid binding protein 4; *Fasn*, fatty acid synthase; *Fgf*, fibroblast growth factor; *Fos*, proto-oncogene c-Fos; *Gapdh*, glyceraldehyde-3-phosphate dehydrogenase; *Gata 3*, GATA-binding factor 3; *Ghr,* growth hormone receptor; *Glut*, glucose transporter; *Ghr*, growth hormone receptor; *Hsl*, hormone sensitive lipase (Lipe); *Hprt1*, hypoxanthine phosphoribosyltransferase 1; *Il1r1*, interleukin 1 receptor type 1; *Il6r*, interleukin 6 receptor alfa; *Insr*, insulin receptor; *Irs*, insulin receptor substrate; *Jun*, jun proto-oncogen; *Klf2*, Kruppel-like factor 2; *Ldha*, lactate dehydrogenase A; *Lep*, leptin; *Lpl*, lipoprotein lipase; *Ncor,* nuclear receptor corepressor; *Nmur1*, neuromedin U receptor 1; *Ntsr1*, neurotensin receptor 1; *Ppar*, peroxisome proliferator-activated receptor; *Ppargc1b*, pparg-gamma coactivator 1 beta; *Ramp3*, receptor activity modifying protein 3; *Retn*, resistin; *Rplp1(13a)*, ribosomal protein p1(13a); *Runx1t1*, runt-related transcription factor 1; *Sfrp1*, secreted frizzled-related protein 1; *Sirt*, sirtuin; *Sort*, sortilin; *Srebf1*, sterol regulatory element binding transcription factor 1; *Stat5a*, signal transducer and activator of transcription 5A; *Thrb*, thyroid hormone receptor, beta; *Tnf*, tumor necrosis factor; *Vegfa*, vascular endothelial growth factor A; *Wnt*, wingless-type MMTV integration site family member.
