# Supplementary material for: Metabolic Disturbances in Rat Sublines with Constitutionally Altered Serotonin Homeostasis
Source: Int J Mol Sci. 2021 May 20;22(10):5400. doi: 10.3390/ijms22105400 (PMC8161318; doi:10.3390/ijms22105400)
Supplement: Supplementary file 1 [file ijms-22-05400-s001.zip › ijms-1233269-SI.pdf]

SUPPLEMENTARY MATERIAL

Supplementary Figure 1

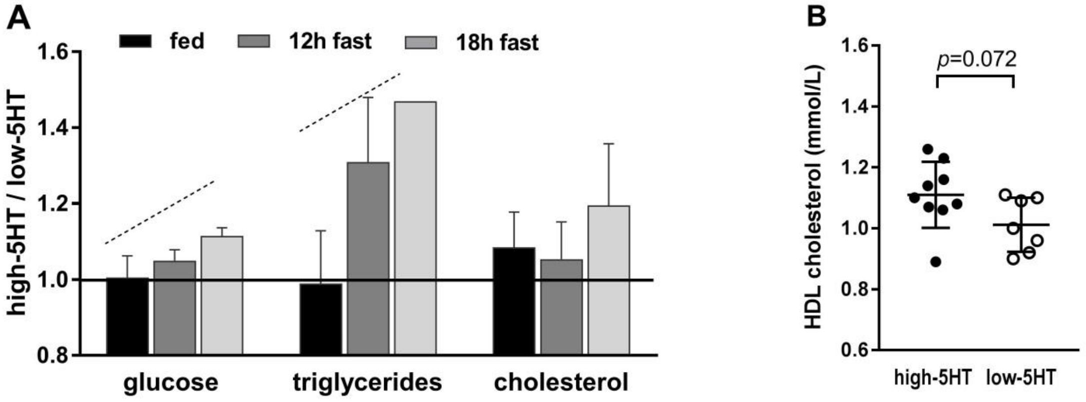

Figure S1. (A) Ratio of blood metabolic parameters between high-5HT and low-5HT subline (H/L) in relation to feeding status of animals. Data represent means ± SD of 2-4 experiments, each with 6-9 animals per subline (except triglycerides 18h fast). **(B)** Serum high-density lipoprotein (HDL) cholesterol levels in high-5HT and low-5HT subline (for total cholesterol levels in the same animals see Figure 2 in the main text). Data are given as individual values and mean ± SD, with *p*-values indicated.

Supplementary Figure 2

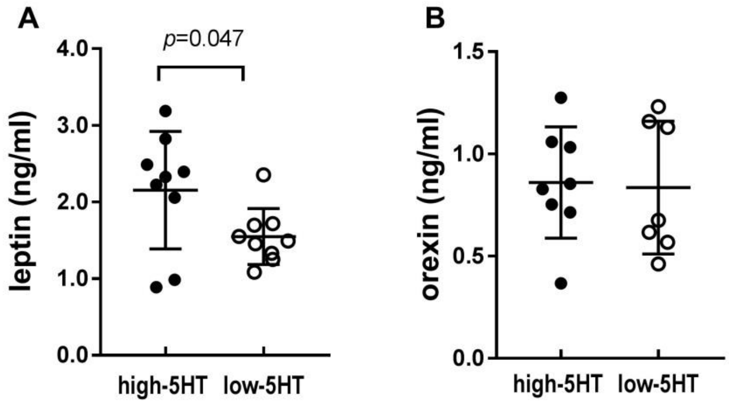

Figure S2. Plasma levels of **(A)** leptin and **(B)** orexin in male high-5HT and low-5HT rats measured in fed state of the animals. Data are presented as individual values and mean ± SD. n=7-9 per group, *p*-values are indicated.

Supplementary Figure 3

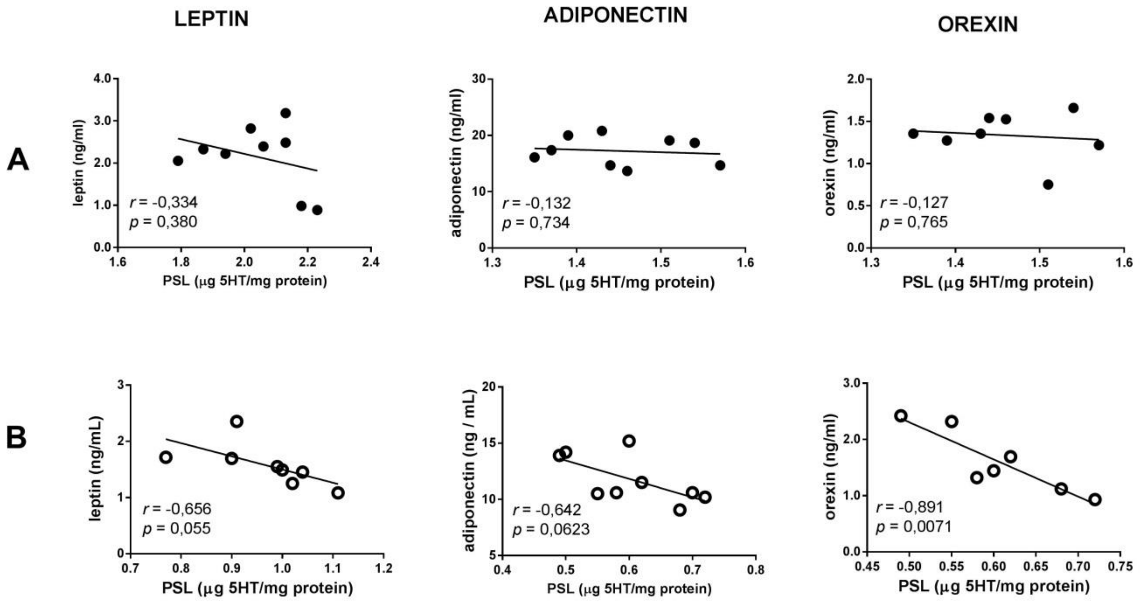

Figure S3. Relationship between platelet serotonin level (PSL) and plasma levels of adipokines: leptin (left), adiponectin (middle) and orexin (right) in (A) high-5HT animals and (B) low-5HT animals. *r*= correlation coefficient, *p*-values are indicated. Negative correlations (significant or trend) were observed only in animals from low-5HT subline.

Supplementary Figure 4

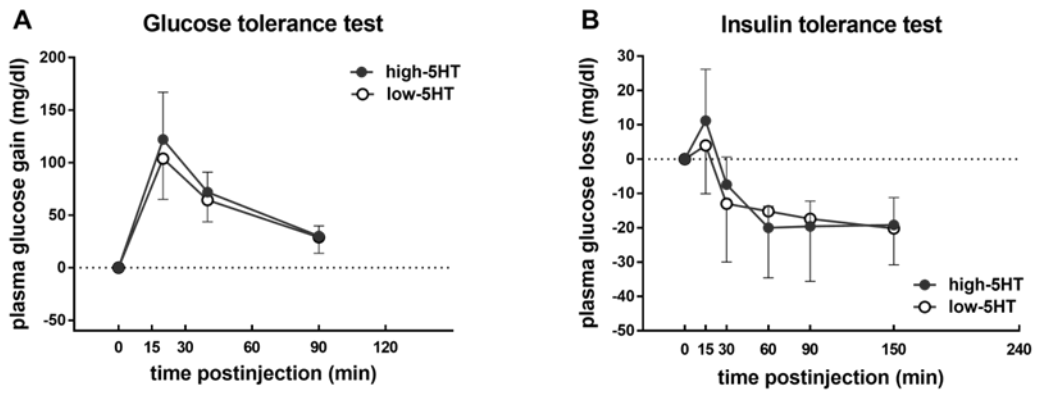

Figure S4. (A) Time course of plasma glucose gain following glucose administration to 2-months old animals from high-5HT and low-5HT sublines subjected to overnight fasting. Presented are means ± SD in groups of 8-9 animals per subline. **(B)** Time course of plasma glucose loss following insulin administration in the same groups of animals as above, after 4 hours fasting. Insulin tolerance test was performed 48 hours after glucose tolerance test. No differences in glucose and insulin tolerance were observed between 5HT sublines.

Supplementary Figure 5

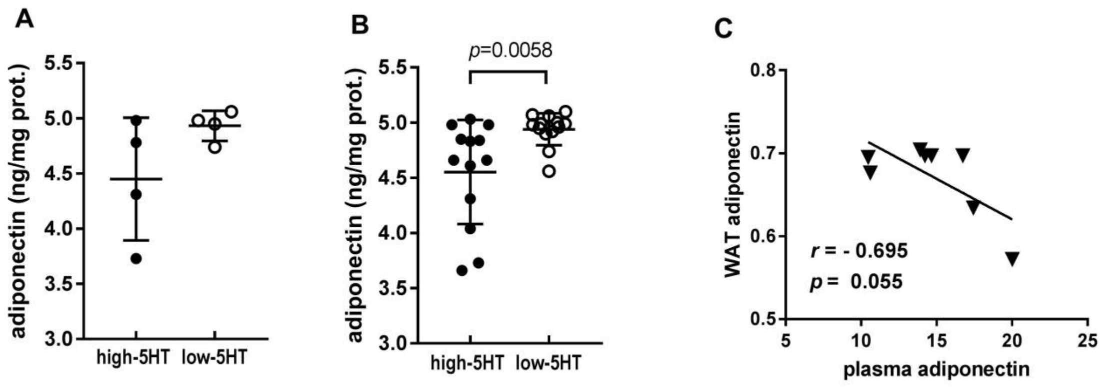

Figure S5. (A) Adiponectin concentration in white adipose tissue (WAT) of high-5HT and low-5HT animals aging 9.5 months and **(B)** pooled values of adiponectin levels measured in animals aged 9.5-months (n, 4 per subline, data from Figure S5A) and 4.5-months (n, 9 per subline, data from Figure 5A in the main text). Data are individual values with indicated mean ± SD, *p*-values are indicated. **(C)** Correlation of adiponectin levels in plasma and white adipose tissue (log transformed values). n=4 animals per subline, *r*=correlation coefficient.

Supplementary Table 1

Table S1. List of genes analysed in visceral white adipose tissue of animals from WZ-5HT sublines using the Rat Adipogenesis RT^2 Profiler PCR Array. Cq = quantification cycle; RER (H/L) = relative expression ratio (high-5HT/low-5HT subline).

Symbol of Gene	Description	Quantification cycle (Cq)		RER (H/L)
		high-5HT	low-5HT	
Acacb	Acetyl-Coenzyme A carboxylase beta	28.01	28.12	0.98
Adipoq	Adiponectin, C1Q and collagen domain containing	15.98	16.84	1.65
Adrb2	Adrenergic, beta-2-, receptor, surface	26.03	25.71	0.73
Agt	Angiotensinogen (serpin peptidase inhibitor, clade A, member 8)	22.06	21.72	0.72
Angpt2	Angiopoietin 2	25.08	24.89	0.80
Axin1	Axin 1	26.50	26.47	0.89
Bmp2	Bone morphogenetic protein 2	27.20	27.61	1.20
Bmp4	Bone morphogenetic protein 4	25.13	25.30	1.02
Bmp7	Bone morphogenetic protein 7	28.65	28.72	0.95
Ccnd1	Cyclin D1	25.31	24.88	0.67
Cdk4	Cyclin-dependent kinase 4	24.36	24.24	0.84
Cdkn1a	Cyclin-dependent kinase inhibitor 1A	25.71	26.06	1.16
Cdkn1b	Cyclin-dependent kinase inhibitor 1B	27.53	27.23	0.74
Cebpa	CCAAT/enhancer binding protein (C/EBP), alpha	23,38	24.03	1.42
Cebpb	CCAAT/enhancer binding protein (C/EBP), beta	21.57	22.10	1.31
Cebpd	CCAAT/enhancer binding protein (C/EBP), delta	26.75	26.19	0.61
Cfd	Complement factor D (adipsin)	16.42	16.95	1.32
Creb1	CAMP responsive element binding protein 1	25.53	25.41	0.84
Ddit3	DNA-damage inducible transcript 3	23.58	24.15	1.35
Dio2	Deiodinase, iodothyronine, type II	30.55	31.92	n/a
Dkk1	Dickkopf homolog 1 (Xenopus laevis)	35.36	39.41	n/a
Dlk1	Delta-like 1 homolog (Drosophila)	29.56	31.37	n/a
E2f1	E2F transcription factor 1	27.18	27.59	1.21
Egr2	Early growth response 2	30.18	28.74	n/a
Fabp4	Fatty acid binding protein 4, adipocyte	15.13	16.17	1.87
Fasn	Fatty acid synthase	20.01	20.77	1.54
Fgf1	Fibroblast growth factor 1	23.60	23.89	1.11
Fgf10	Fibroblast growth factor 10	26.44	27.08	1.41
Fgf2	Fibroblast growth factor 2	29.65	29.95	1.12
Fos	FBJ osteosarcoma oncogene	25.41	25.04	0.70
Foxc2	Forkhead box C2	28.19	28.42	1.07
Gata2	GATA binding protein 2	26.91	27.04	0.99
Gata3	GATA binding protein 3	28.43	29.18	1.52
Hes1	Hairy and enhancer of split 1 (Drosophila)	24.97	24.93	0.88
Insr	Insulin receptor	25.19	25.32	0.99
Irs1	Insulin receptor substrate 1	24.73	24.29	0.67
Irs2	Insulin receptor substrate 2	24.06	24.45	1.19
Jun	Jun oncogene	23.42	23.15	0.75
Klf15	Kruppel-like factor 15	25.29	25.23	0.87
Klf2	Kruppel-like factor 2 (lung)	29.90	29.53	0.70
Klf3	Kruppel-like factor 3 (basic)	23.52	23.55	0.92
Klf4	Kruppel-like factor 4 (gut)	23.02	22.93	0.86
Lep	Leptin	20.40	21.68	2.20

Lipe	Lipase, hormone sensitive	20.38	21.16	1.55
Lmna	Lamin A	23.02	22.85	0.81
Tcf7l2	Transcription factor 7-like 2 (T-cell specific, HMG-box)	24.29	24.27	0.90
Lpl	Lipoprotein lipase	18.05	18.72	1.44
Lrp5	Low density lipoprotein receptor-related protein 5	25.32	25.41	0.97
Mapk14	Mitogen activated protein kinase 14	25.40	25.36	0.88
Ncoa2	Nuclear receptor coactivator 2	24.77	24.87	0.97
Ncor1	Nuclear receptor co-repressor 1	24.59	25.13	1.31
Ncor2	Nuclear receptor co-repressor 2	27.73	27.30	0.68
Nr0b2	Nuclear receptor subfamily 0, group B, member 2	34.12	31.15	n/a
Nr1h3	Nuclear receptor subfamily 1, group H, member 3	24.07	24.30	1.07
Nrf1	Nuclear respiratory factor 1	27.02	26.91	0.84
Ppara	Peroxisome proliferator activated receptor alpha	25.94	26.14	1.04
Ppard	Peroxisome proliferator-activated receptor delta	26.22	26.29	0.95
Pparg	Peroxisome proliferator-activated receptor gamma	22.36	23.15	1.57
Ppargc1a	Peroxisome proliferator-activated receptor gamma, coactivator 1 alpha	30.11	30.86	n/a
Ppargc1b	Peroxisome proliferator-activated receptor gamma, coactivator 1 beta	26.33	27.19	1.65
Rb1	Retinoblastoma 1	24.73	25.25	1.30
Retn	Resistin	19.41	20.46	1.89
Runx1t1	Runt-related transcription factor 1; translocated to, 1 (cyclin D-related)	27.92	27.54	0.70
Rxra	Retinoid X receptor alpha	23.45	23.66	1.05
Sfrp1	Secreted frizzled-related protein 1	24.16	22.91	0.38
Sfrp5	Secreted frizzled-related protein 5	29.80	29.54	0.76
Shh	Sonic hedgehog	33.12	32.41	n/a
Sirt1	Sirtuin (silent mating type information regulation 2 homolog) 1	24.79	24.57	0.78
Sirt2	Sirtuin (silent mating type information regulation 2 homolog) 2	27.32	26.91	0.68
Sirt3	Sirtuin (silent mating type information regulation 2 homolog) 3	26.66	27.02	1.17
Slc2a4	Solute carrier family 2 (facilitated glucose transporter), member 4	23.14	23.64	1.28
Src	V-src sarcoma (Schmidt-Ruppin A-2) viral oncogene homolog	28.13	27.99	0.82
Srebf1	Sterol regulatory element binding transcription factor 1	25.67	26.26	1.36
Stat5a	Signal transducer and activator of transcription 5A	26.31	26.96	1.42
Taz	Tafazzin	25.72	25.97	1.07
Tsc22d3	TSC22 domain family, member 3	23.49	23,30	0.80
Twist1	Twist homolog 1 (Drosophila)	26.02	26.26	1.07
Ucp1	Uncoupling protein 1 (mitochondrial, proton carrier)	34.12	36.32	n/a
Vdr	Vitamin D (1,25- dihydroxyvitamin D3) receptor	29.41	29.77	1.16
Wnt1	Wingless-type MMTV integration site family, member 1	32.20	33.02	n/a
Wnt10b	Wingless-type MMTV integration site family, member 10B	29.17	29.93	1.55
Wnt3a	Wingless-type MMTV integration site family, member 3A	29.20	30.29	n/a
Wnt5a	Wingless-type MMTV integration site family, member 5A	25.62	25.47	0.82
Wnt5b	Wingless-type MMTV integration site family, member 5B	27.86	27.64	0.78
Rplp1	Ribosomal protein, large, P1	18.26	18.30	n/a
Hprt1	Hypoxanthine phosphoribosyltransferase 1	24.08	24.20	n/a
Rpl13a	Ribosomal protein L13A	20.07	20.07	n/a
Ldha	Lactate dehydrogenase A	21.78	22.36	n/a
Actb	Actin, beta	19.10	19.08	n/a

Supplementary Table 2

Table S2. List of genes analysed in visceral white adipose tissue of animals from WZ-5HT sublines using the Rat Obesity RT^2 Profiler PCR Array. Cq = quantification cycle, RER(H/L) = relative expression ratio (high-5HT/low-5HT subline).

Symbol of Gene	Description	Quantification cycle (Cq)		RER (H/L)
		high-5HT	low-5HT	
Adcyap1	Adenylate cyclase activating polypeptide 1	33.61	33.69	n/a
Adcyap1r1	Adenylate cyclase activating polypeptide 1 receptor 1	27.72	27.58	0.84
Adipoq	Adiponectin, C1Q and collagen domain containing	16.05	16.78	1.53
Adipor1	Adiponectin receptor 1	23.15	23.73	1.39
Adipor2	Adiponectin receptor 2	24.04	24.74	1.51
Adra2b	Adrenergic, alpha-2B-, receptor	31.91	32.43	n/a
Adrb1	Adrenergic, beta-1-, receptor	26.58	27.85	2.24
Agrp	Agouti related protein homolog (mouse)	30.37	31.01	n/a
Apoa4	Apolipoprotein A-IV	36.23	26.59	n/a
Atrn	Attractin	26.24	26.65	1.24
Bdnf	Brain-derived neurotrophic factor	n.d.	33.41	n/a
Brs3	Bombesin-like receptor 3	n.d.	n.d.	n/a
C3	Complement component 3	31.34	24.39	n/a
Calca	Calcitonin-related polypeptide alpha	30.01	31.11	n/a
Calcr	Calcitonin receptor	33.24	34.02	n/a
Cartpt	CART prepropeptide	36.27	35.72	n/a
Cck	Cholecystokinin	35.88	33.65	n/a
Cckar	Cholecystokinin A receptor	33.51	34.47	n/a
Clps	Colipase, pancreatic	32.24	35.23	n/a
Cnr1	Cannabinoid receptor 1 (brain)	30.51	31.91	n/a
Cntf	Ciliary neurotrophic factor	26.74	28.16	2.48
Cntfr	Ciliary neurotrophic factor receptor	29.00	28.22	0.54
Crh	Corticotropin releasing hormone	n.d.	n.d.	n/a
Crhr1	Corticotropin releasing hormone receptor 1	n.d.	n.d.	n/a
Drd1a	Dopamine receptor D1A	33.08	33.94	n/a
Drd2	Dopamine receptor D2	33.79	33.00	n/a
Gal	Galanin prepropeptide	32.35	31.56	n/a
Galr1	Galanin receptor 1	31.74	31.55	n/a
Gcg	Glucagon	n.d.	32.32	n/a
Gcgr	Glucagon receptor	31.33	28.58	n/a
Gh1	Growth hormone 1	37.85	33.53	n/a
Ghr	Growth hormone receptor	21.10	22.04	1.78
Ghrl	Ghrelin/obestatin prepropeptide	29.58	29.71	1.02
Ghsr	Growth hormone secretagogue receptor	34.96	33.79	n/a
Glp1r	Glucagon-like peptide 1 receptor	34.67	36.23	n/a
Prlhr	Prolactin releasing hormone receptor	n.d.	n.d.	n/a
Mchr1	Melanin-concentrating hormone receptor 1	33.12	32.05	n/a
Grp	Gastrin releasing peptide	n.d.	37.98	n/a
Grpr	Gastrin releasing peptide receptor	37.47	34.78	n/a
HcRt	Hypocretin	35.60	n.d.	n/a
Hcrtr1	Hypocretin (orexin) receptor 1	32.97	32.02	n/a
Hrh1	Histamine receptor H 1	30.87	30.52	n/a

Htr2c	5-hydroxytryptamine (serotonin) receptor 2C	32.43	32.16	n/a
Iapp	Islet amyloid polypeptide	n.d.	34.49	n/a
Il1a	Interleukin 1 alpha	n.d.	30.82	n/a
Il1b	Interleukin 1 beta	30.50	28.65	n/a
Il1r1	Interleukin 1 receptor, type I	26.56	25.68	0.51
Il6	Interleukin 6	33.42	32.24	n/a
Il6r	Interleukin 6 receptor	25.80	25.04	0.55
Ins1	Insulin 1	35.44	34.03	n/a
Ins2	Insulin 2	31.72	33.97	n/a
Insr	Insulin receptor	25.14	25.51	1.20
Lep	Leptin	20.69	22.22	2.69
Lepr	Leptin receptor	31.72	31.30	n/a
Mc3r	Melanocortin 3 receptor	38.62	n.d.	n/a
Nmb	Neuromedin B	27.75	27.68	0.89
Nmbr	Neuromedin B receptor	33.14	32.04	n/a
Nmu	Neuromedin U	34.07	32.32	n/a
Nmur1	Neuromedin U receptor 1	29.40	29.00	0.70
Npy	Neuropeptide Y	27.33	27.15	0.82
Npy1r	Neuropeptide Y receptor Y1	26.84	26.60	0.78
Nr3c1	Nuclear receptor subfamily 3, group C, member 1	23.72	24.15	1.25
Ntrk1	Neurotrophic tyrosine kinase, receptor, type 1	36.78	38.09	n/a
Nts	Neurotensin	34.02	n.d.	n/a
Ntsr1	Neurotensin receptor 1	28.98	28.33	0.59
Oprk1	Opioid receptor, kappa 1	36.26	33.38	n/a
Oprm1	Opioid receptor, mu 1	34.56	33.32	n/a
Sigmar1	Sigma non-opioid intracellular receptor 1	24.57	24.41	0.83
Pomc	Proopiomelanocortin	26.54	27.34	1.61
Ppara	Peroxisome proliferator activated receptor alpha	26.57	27.02	1.26
Pparg	Peroxisome proliferator-activated receptor gamma	22.76	23.52	1.57
Ppargc1a	Peroxisome proliferator-activated receptor gamma, coactivator 1 alpha	30.67	31.50	n/a
Ptpn1	Protein tyrosine phosphatase, non-receptor type 1	26.13	26.33	1.06
Pyy	Peptide YY (mapped)	35.58	34.08	n/a
Ramp3	Receptor (G protein-coupled) activity modifying protein 3	29.67	28.39	0.38
Sort1	Sortilin 1	23.01	23.78	1.57
Sst	Somatostatin	35.09	36.08	n/a
Sstr1	Somatostatin receptor 1	32.16	32.85	n/a
Thrb	Thyroid hormone receptor beta	25.05	25.62	1.38
Tnf	Tumor necrosis factor (TNF superfamily, member 2)	30.78	31.23	n/a
Trh	Thyrotropin releasing hormone	n.d.	32.93	n/a
Trhr	Thyrotropin releasing hormone receptor	35.26	35.05	n/a
Ucn	Urocortin	39.48	36.11	n/a
Ucp1	Uncoupling protein 1 (mitochondrial, proton carrier)	34.84	35.73	n/a
Rplp1	Ribosomal protein, large, P1	18.02	17.92	n/a
Hprt1	Hypoxanthine phosphoribosyltransferase 1	23.39	23.34	n/a
Rpl13a	Ribosomal protein L13A	19.87	19.84	n/a
Ldha	Lactate dehydrogenase A	21.36	22.03	n/a
Actb	Actin, beta	18.48	18.53	n/a

Supplementary Table 3

Table S3. Primer sequences used in RT-qPCR analysis

NCBI symbol	Gene	Forward primer sequence	Reverse primer sequence
Actb	Actin beta	5'-GCGCAAGTACTCTGTGTGGA	5'-ACATCTGCTGGAAGGTGGAC
Adipoq	Adiponectin	5'-GAGACGCAGGTGTTCTTG	5'-CCTACGCTGAATGCTGAG
Adipor1	Adiponectin receptor 1	5'-GCTGGCCTTTATGCTGCTCG	5'-TCTAGGCCGTAACGGAATTC
Adipor2	Adiponectin receptor 2	5'-TCTAG CCGTAACGGAATTC	5'-GATACTGAGGGGTGGCAAAC
Atgl	Adipose triglyceride lipase	5'-AGACTGTCTGAGCAGGTGGA	5'-AGTAGCTGACGCTGGCATTC
Cebpa	CCAAT enhancer binding protein alpha	5'-GACCATCCGCCTTGTGTGTA	5'-CTGACATTGCACAAGGCACC
Cebpb	CCAAT/enhancer binding protein beta	5'-GACAAGCTGAGCGACGAGTA	5'-AGCTGCTCCACCTTCTTCTG
Cebpd	CCAAT/enhancer binding protein delta	5'-GAATTGCTACAGTTTCTTGG	5'-ATGCGCAGTCTCTTCCTC
Cfd	Complement factor D	5'-CCTACATGGCTTCAGTGCAA	5'-TTCAGGACTGGACAGGGAGT
Fabp4	Fatty acid binding protein 4	5'-AGAAGTGGGAGTTGGCTTCG	5'-ACTCTCTGACCGGATGACGA
Fasn	Fatty acid synthase	5'-GGTAGGCTTGGTGAACTGTCTC	5'-TCTAACTGGAAGTGACGGAAGG
Fgf2	Fibroblast growth factor 2	5'-TTCACAGCCTGTGCTCTAGGG	5'-GATCGGGTCAGGTTTTGGAAA
Fgf10	Fibroblast growth factor 10	5'-GAGATGTCCGCTGGAGAAAG	5'-CCCCTTCTTGTTCATGGCTA
Fgf21	Fibroblast growth factor 21	5'-AGGCTTTGACACCCAGGATT	5'-ACAGATGACGACCAGGACAC
Gapdh	Glyceraldehyde-3-phosphate dehydrogenase	5'-TGCCCCCATGTTTGTGATG	5'-TGGTGGTGCAGGATGCATT
Glut1	Glucose transporter 1	5'-TGGCCAAGGACACACGAATACTGA	5'-TGGAAGAGACAGGAATGGGCGAAT
Glut4	Glucose transporter 4	5'-ATCAACGCCCCACAGAAAGT	5'-CCTGCCTACCCAGCCAAGT
Hsl	Hormone-sensitive lipase	5'-CTCCTCATGGCTCAACTCC	5'-ACTCCTGCGCATAGACTCC
Insr	Insulin receptor	5'-ATCTCCTGGGATTCATGCTG	5'-TACTGGGTCCAGGGTTTGAG
Irs1	Insulin receptor substrate 1	5'-GATTTAAGCACCTATGCCAG	5'-GAATCGTGAAAGAGTTCGAG
Irs2	Insulin receptor substrate 2	5'-CCACACACCTGTCCTCATTG	5'-TAATCCGCTTTGCCAAAATC
Lep	Leptin	5'-GAC ACC AAA ACC CTC AT	5'-CAG GGT CTG GTC CAT CT
Lpl	Lipoprotein lipase	5'-TTGAGAAAGGGCTCTGCCTGAGTT	5'-TGCTTCTCTTGGCTCTGACCTTGT
Pparg	Peroxisome proliferator activated receptor gamma	5'-TCGCTGATGCACTGCCTATG	5'-TGATTCCGAAGTTGGTGGGC
Ppargc1b	Peroxisome proliferative activated receptor, gamma, coactivator 1 beta	5'-CTACCAGAGCCCACCCAGTA	5'-CAGGATGAGGAGCCAGAACT
Retn	Resistin	5'-ACT TCA GCT CCC TAC TG	5'-GTC TAT GCT TCC GCA CT
Tnf	Tumor necrosis factor	5'-CATCTTCTCAAAACTCGAGTGACAA	5'-TGGGAGTAGATAAGGTACAGCCC
Vegfa	Vascular endothelial growth factor A	5'-CCGGTTTAAATCCTGGAGCG	5'-TTTAACTCAAGCTGCCTCGC
